# Supplementary material for: A unified approach for identifying PET-based neuronal activation and molecular connectivity with the functional PET toolbox
Source: J Cereb Blood Flow Metab. 2025 Sep 8;46(2):558–68. doi: 10.1177/0271678X251370831 (PMC12417444; doi:10.1177/0271678X251370831)

SUPPLEMENTARY MATERIAL for

A Unified Approach for Identifying PET-based Neuronal Activation and Molecular Connectivity with the functional PET toolbox

Andreas Hahn[1,2 #], Murray B. Reed[1,2], Christian Milz[1,2], Pia Falb[1,2], Matej Murgaš[1,2], Rupert Lanzenberger[1,2 #]

[1] *Department of Psychiatry and Psychotherapy, Medical University of Vienna, Vienna, Austria*

[2] *Comprehensive Center for Clinical Neurosciences and Mental Health (C3NMH), Medical University of Vienna, Vienna, Austria*

SUPPLEMENTARY METHODS

Example demonstration of the graphical user interface (GUI)

The subsequent figures provide a step-by-step guide for an example GLM analysis to identify task effects in a simulated fPET data set.

First, add the fPET toolbox (add with subfolders) and SPM12 to the Matlab path. Then start the fPET toolbox with the following command:

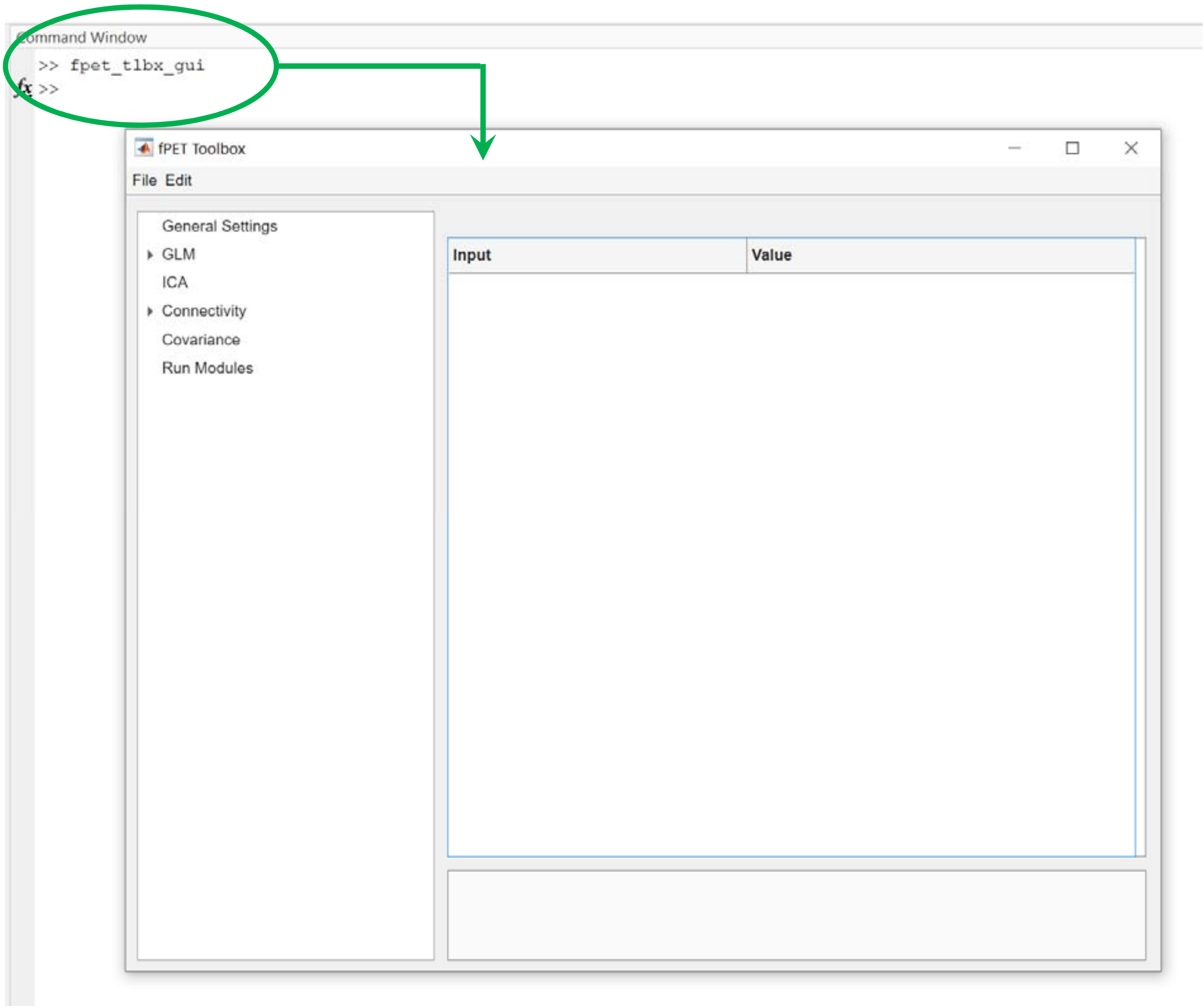

In the toolbox GUI the sections on the left describe the different analyses. Clicking on one of them shows the detail options in the window on the right. Clicking on a specific variable opens a description in the lower right window. Clicking on the field "value" allows to enter the desired value for a particular variable. Mandatory variables are marked with an asterisk.

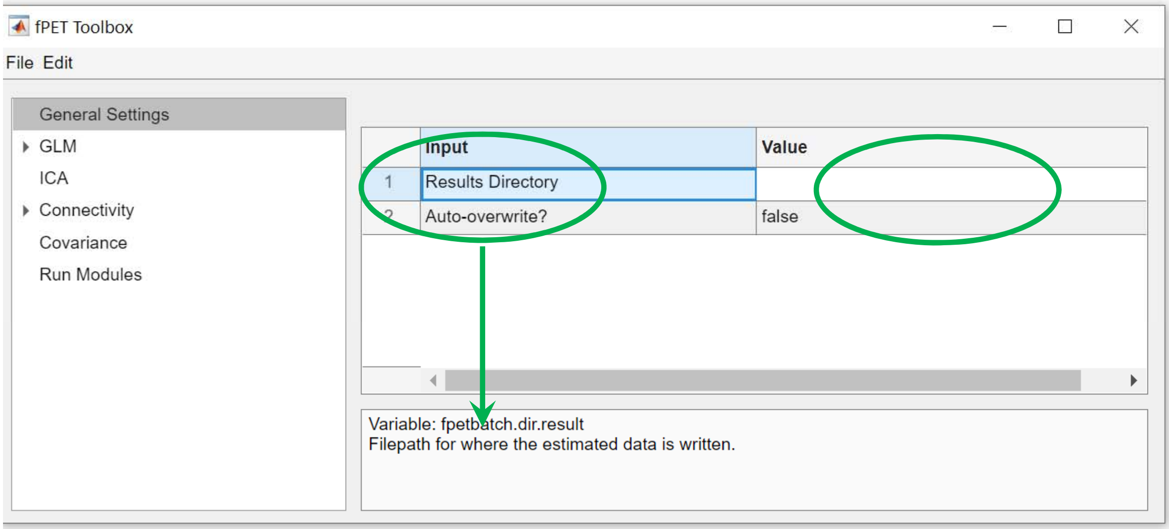

The essential setting to run a certain analysis is to enable the corresponding variable, in this case “Run GLM” needs to be “true”.

The basic settings for a GLM calculation cover the 4D fPET nifti input image, the length of each frame and the specification if GLM temporal inputs are given in seconds or frames.

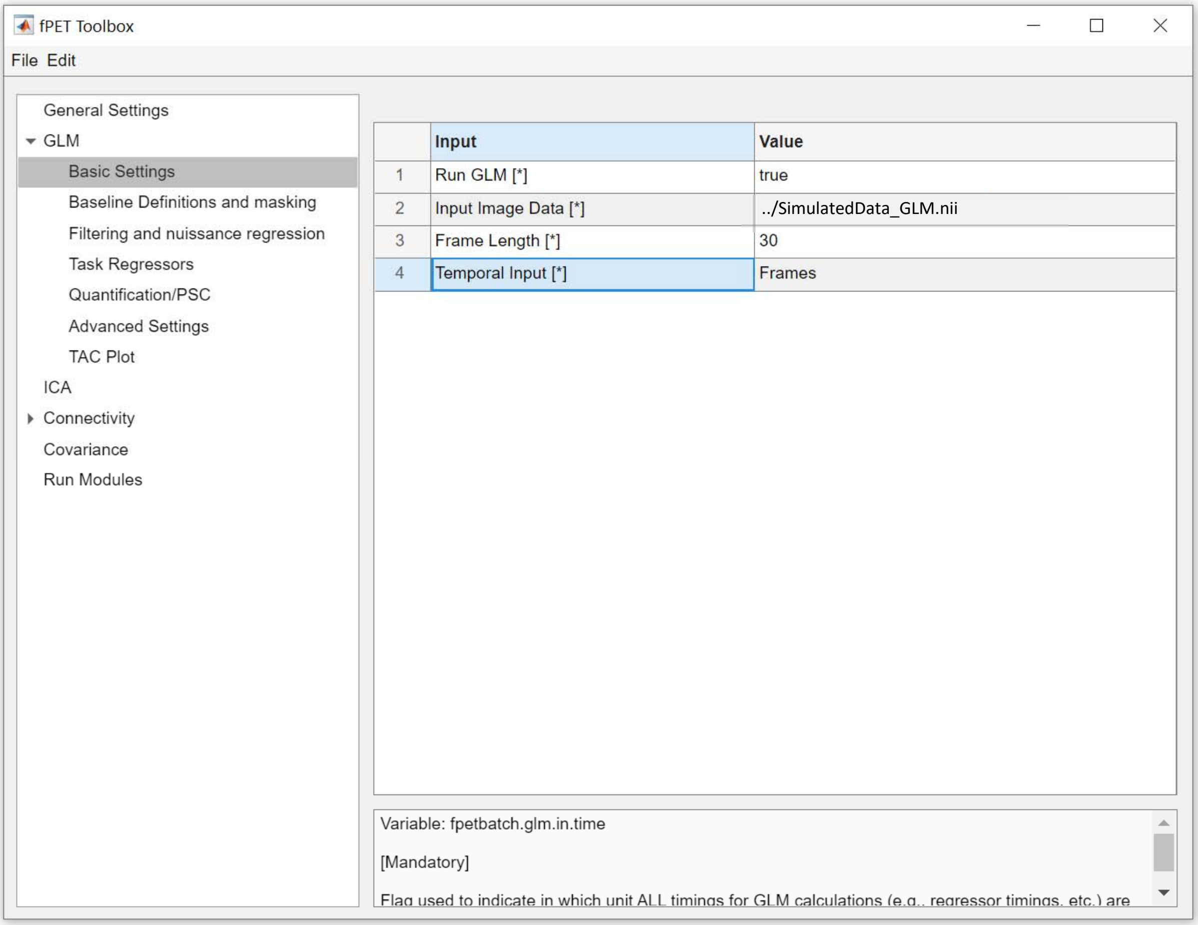

The “baseline mask” is required to extract a representative TAC with baseline radiotracer uptake, usually this is a 3D nifti file covering the gray matter.

The “calculation mask” defines where calculations are actually carried out. Again, this is usually a 3D nifti file covering the gray matter. All other voxels are set to zero, which will speed up the calculation.

If a priori knowledge exists where activation is to be expected, this can be accounted for by providing a “baseline exclusion mask” (3D nifti file). Voxels in this mask will not be used for the extraction of the baseline TAC. As a result, the baseline TAC is not contaminated by task effects.

For the “baseline definition” one can use the extracted baseline TAC as described above (option “mask”, default) or model the baseline TAC with a third order polynomial.

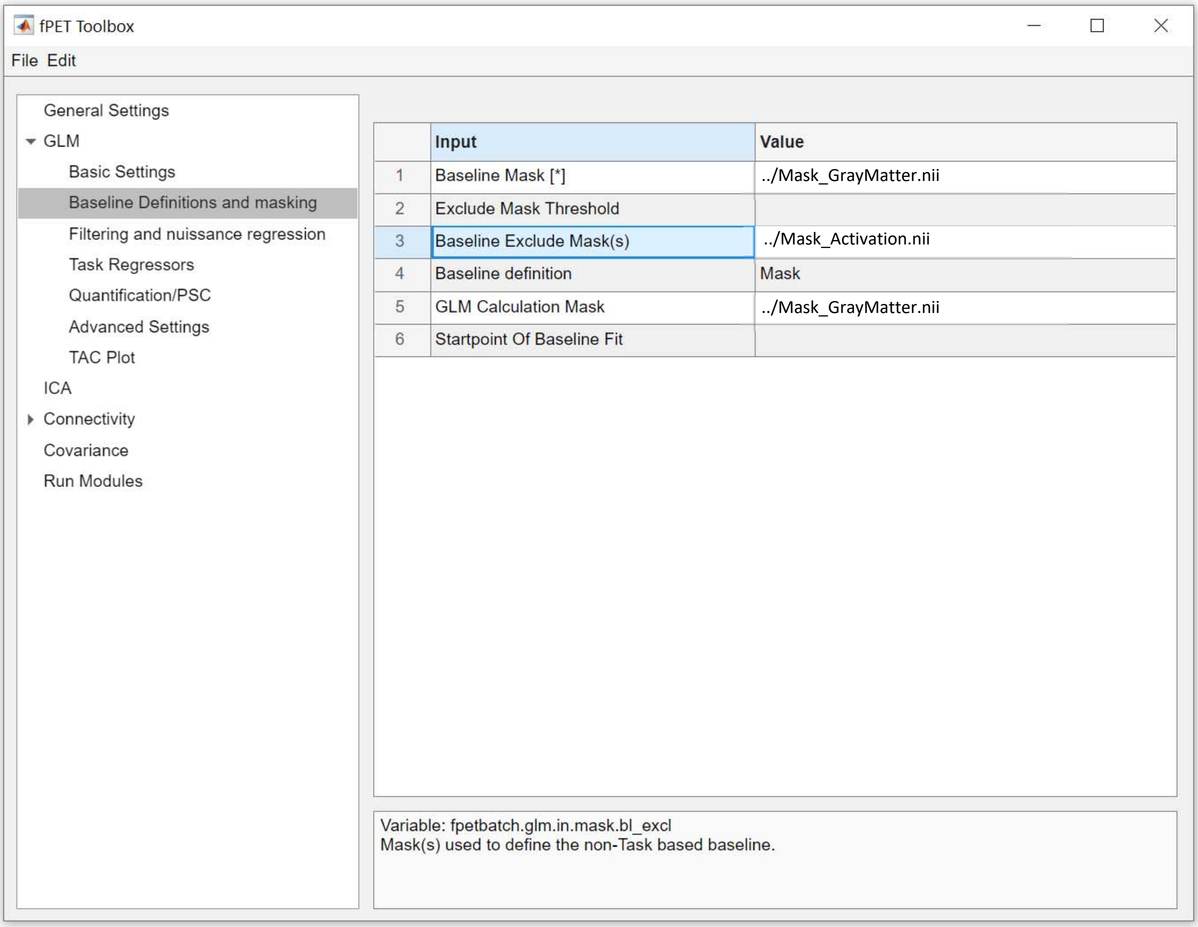

A task regressor is added by entering the name, onset and end of the respective stimulation. As the variable “temporal inputs” above was defined as “frames” this also applies for the task regressor.

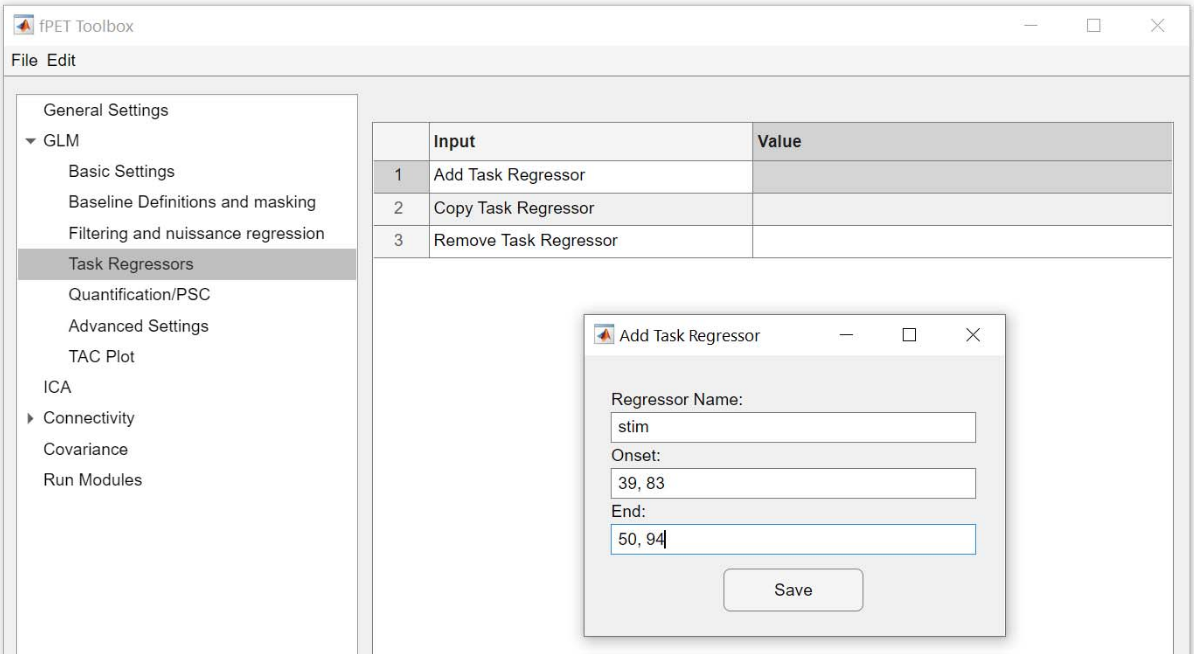

Resulting output:

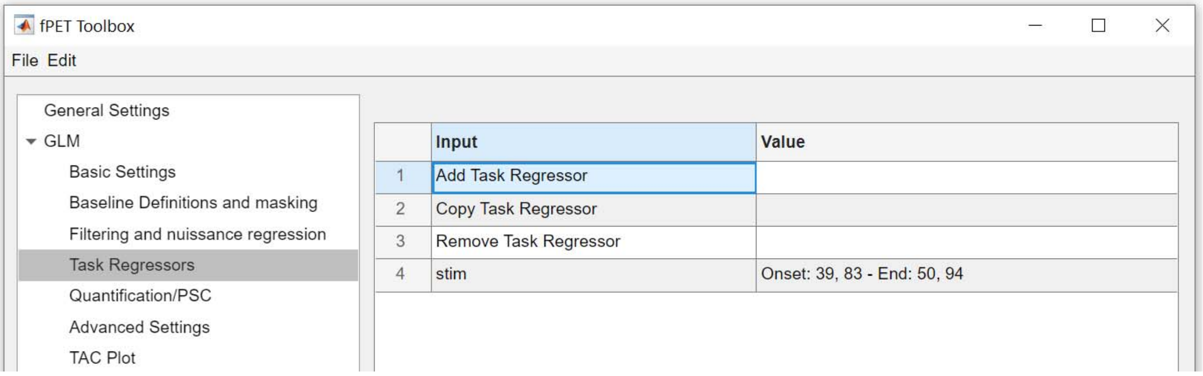

The calculation of percent signal change is recommended for GLM analyses, which is enabled by setting the “Estimate Percent Signal Change” to “true”.

Similarly, absolute quantification is carried out with the variable “Quantify data” being “true”. Here, temporal inputs for blood data needs to be specified (seconds or minutes) and blood input data provided, in this case a whole-blood curve and a plasma input curve. These are text files with two columns, containing time and activity, respectively. If blood glucose levels are provided, the cerebral metabolic rate of glucose (CMRGlu) is calculated in addition to the net influx constant (Ki).

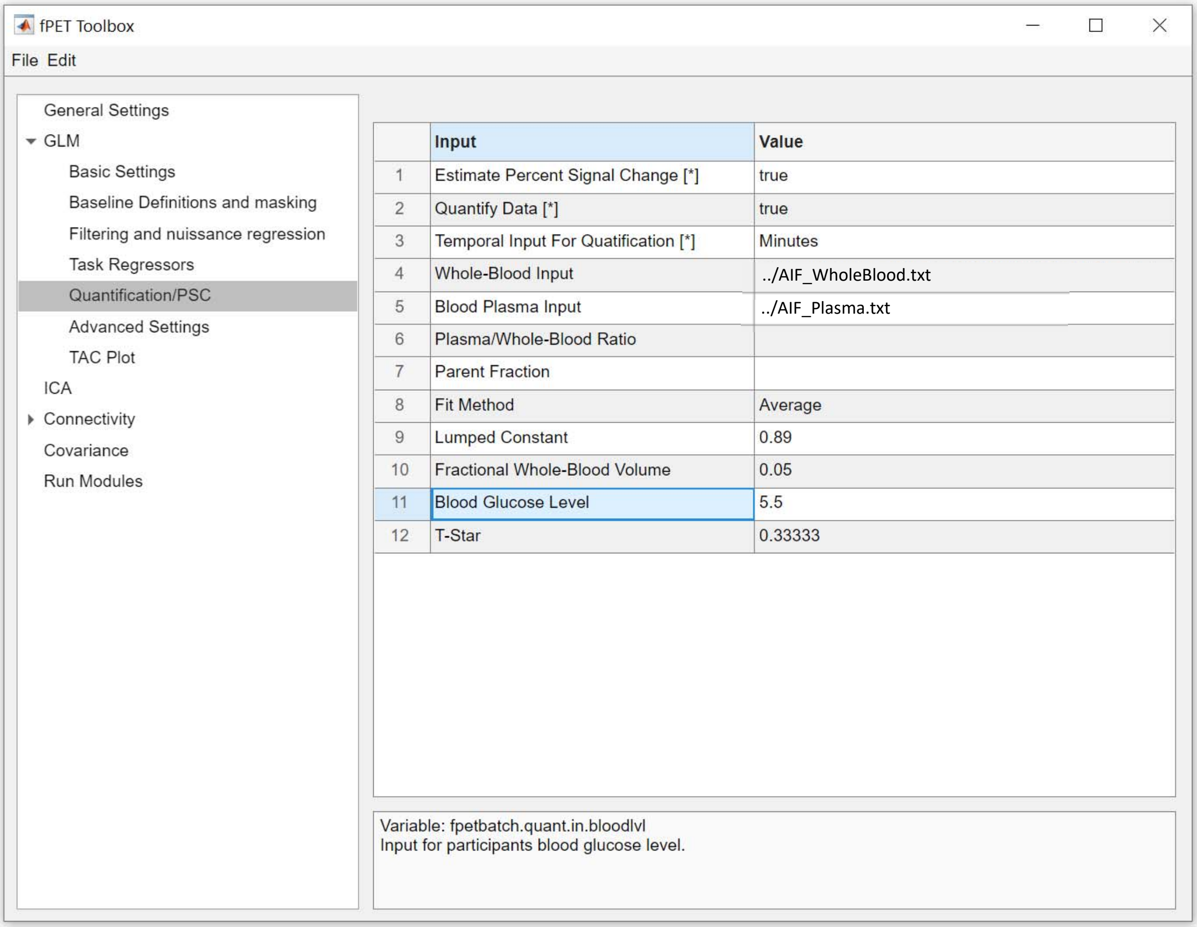

The final page provides an overview of the selected analyses and allows to run the calculations.

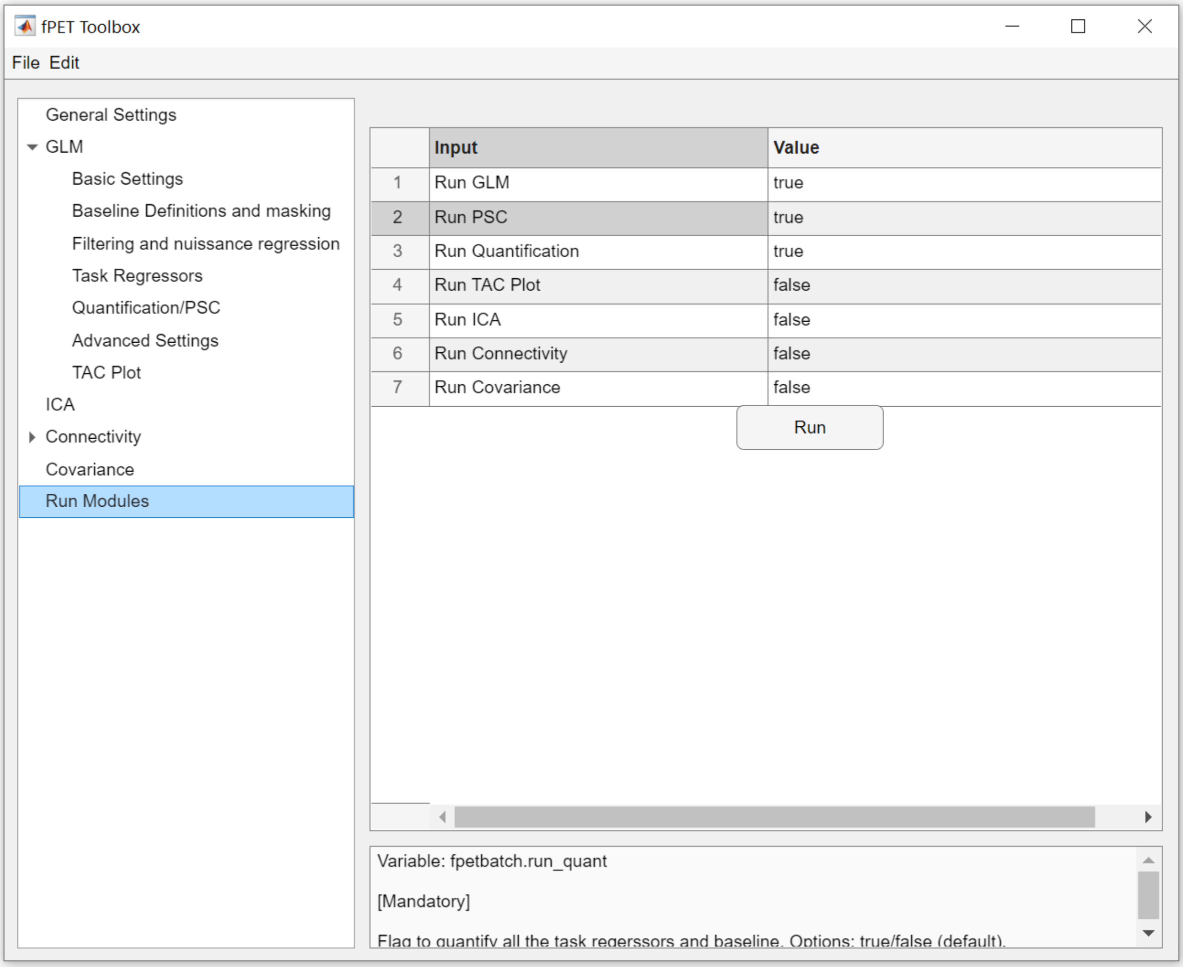

After successful analysis, TACs can be plotted, by setting the “Run TAC plot” to “true”. The obtained file “fPET_glm.mat” is entered as input (or files from multiple subjects if available).

The desired regressors to be plotted needs to be determined. Regressors are saved in the order 1=constant, 2=baseline, 3=stimulation, etc.

Furthermore, the region of interest for the TAC extraction (“TAC mask”) needs to be provided (3D nifti file).

Finally, one can chose to plot TACs for each individual and/or the average across all subjects (baseline and task separately) as well as the raw TACs for the given region.

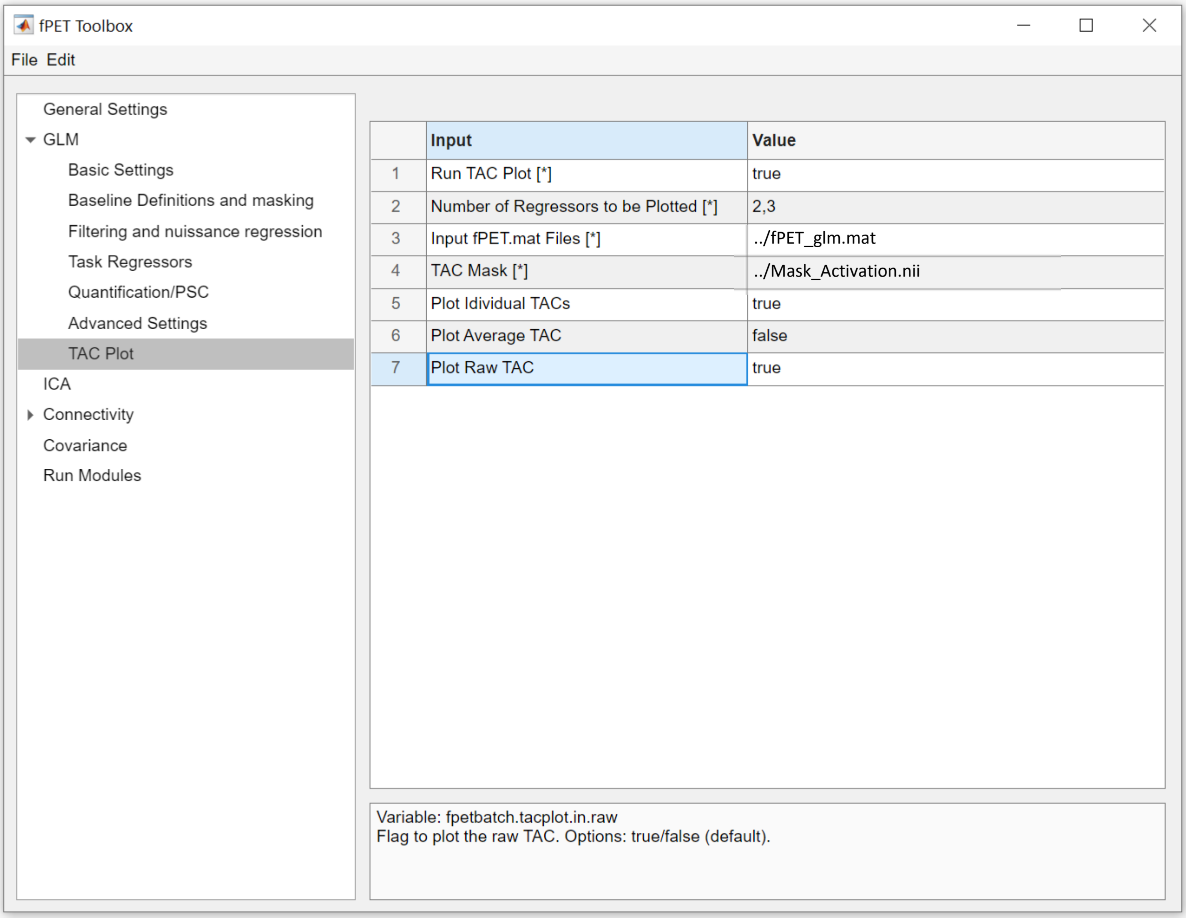

For any of the analyses, the corresponding m-code can be obtained from the GUI, which enables simplified scripting.

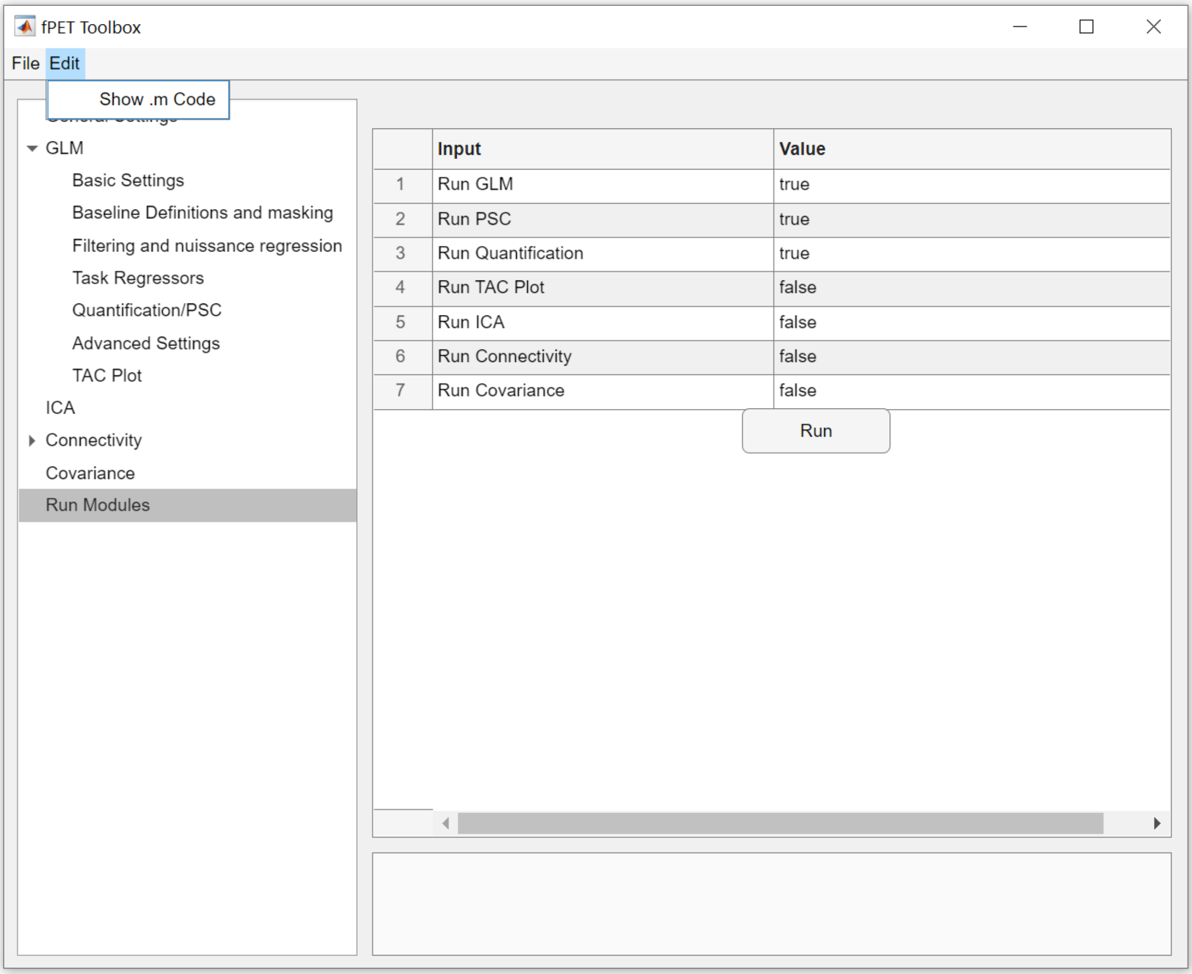

Supplement: sj-pdf-1-jcb-10.1177_0271678X251370831 – Supplemental material for A unified approach for identifying PET-based neuronal activation and molecular connectivity with the functional PET toolbox [file sj-pdf-1-jcb-10.1177_0271678X251370831.pdf]
